# Effect of Obstructive Sleep Apnea and CPAP Treatment on the Bioavailability of Erythrocyte and Plasma Nitric Oxide

**DOI:** 10.3390/ijerph192214719

**Published:** 2022-11-09

**Authors:** Jakub Mochol, Jakub Gawryś, Ewa Szahidewicz-Krupska, Jerzy Wiśniewski, Paulina Fortuna, Piotr Rola, Helena Martynowicz, Adrian Doroszko

**Affiliations:** 1 Clinical Department of Internal and Occupational Diseases, Hypertension and Clinical Oncology, Faculty of Medicine, Wroclaw Medical University, 50-367 Wroclaw, Poland; 2 Department of Biochemistry and Immunochemistry, Faculty of Medicine, Wroclaw Medical University, 50-367 Wroclaw, Poland; 3 Department of Biochemistry, Molecular Biology and Biotechnology, Faculty of Chemistry, Wroclaw University of Science and Technology, 50-370 Wroclaw, Poland; 4 Department of Cardiology, Provincial Specialized Hospital, 59-220 Legnica, Poland

**Keywords:** obstructive sleep apnea, endothelial dysfunction, oxidative stress, nitric oxide, asymmetric dimethylarginine

## Abstract

Introduction: Endothelial dysfunction resulting from decreased nitric oxide (NO) bioavailability is an important mechanism that increases cardiovascular risk in subjects with obstructive sleep apnea (OSA). NO is produced by nitric oxide synthase (NOS) in a reaction that converts L-arginine to L-citrulline. Asymmetric-dimethylarginine (ADMA) is created by L-arginine and is a naturally occurring competitive inhibitor of nitric oxide synthase (NOS). The aim of our study was to verify if erythrocytes could play a role in the storage and accumulation of ADMA in OSA patients. The crosstalk between erythrocyte-ADMA, SDMA, L-arginine, and L-citrulline levels and endothelial function was investigated in OSA subjects both at baseline and prospectively following 1-year CPAP (continuous positive airway pressure) treatment. Material and Methods: A total of 46 subjects with OSA were enrolled in this study and divided into two groups: those with moderate-to-severe OSA and those with mild or no OSA. A physical examination was followed by blood collection for the assessment of biochemical cardiovascular risk factors and the nitric oxide bioavailability parameters both in plasma and erythrocytes. Vasodilative endothelial function was assessed using Laser Doppler Flowmetry (LDF). Results: No significant changes regarding the NO pathway metabolites were noted apart from the plasma L-citrulline concentration, which was decreased in patients with OSA (26.9 ± 7.4 vs. 33.1 ± 9.4 μM, *p* < 0.05). The erythrocyte ADMA concentration was lower than in plasma irrespective of the presence of OSA (0.33 ± 0.12 vs. 0.45 ± 0.08 μM in OSA, *p* < 0.05 and 0.33 ± 0.1 vs. 0.45 ± 0.07 μM in the control, *p* < 0.05). No significant changes regarding the LDF were found. CPAP treatment did not change the levels of NO metabolites in the erythrocytes. Conclusions: The erythrocyte pool of the NO metabolic pathway intermediates does not depend on OSA and its treatment, whereas the erythrocytes could constitute a high-volume buffer in their storage Hence, the results from this prospective study are a step forward in understanding the role of the erythrocyte compartment and the intra-erythrocyte pathways regulating NO bioavailability and paracrine endothelial function in the hypoxia-reoxygenation setting, such as obstructive sleep apnea.

## 1. Introduction

**Figure 1:**
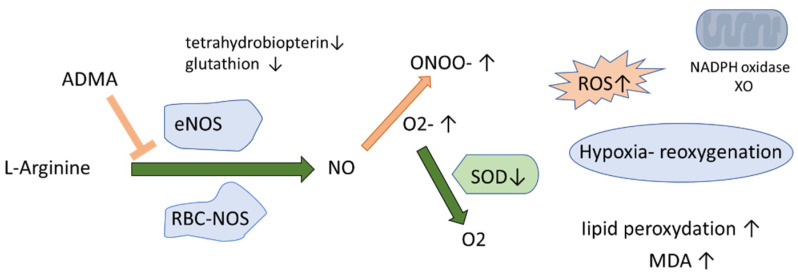
Oxidative stress in OSA. Abbreviations: RBC-NOS: red blood cell nitric oxide synthase; ADMA: asymmetric dimethylarginine SOD: superoxide dismutase; XOD: xanthine oxidase; ONOO^−^: peroxynitrite; O_2_^−^: superoxide; ROS: reactive oxygen species; MDA: malonyldialdehyde; eNOS: endothelial nitric oxide synthase, RBC-NOS: nitric oxide synthase in the erythrocyte.

Obstructive sleep apnea (OSA) is recognized as an independent cardiovascular risk factor and is estimated to affect close to one billion patients worldwide [1]. Increased intra-thoracic pressure, activation of the adrenergic system, increased release of inflammatory cytokines, and oxidative stress, all leading to endothelial dysfunction [2,3] are among the pivotal pathogenic factors that contribute to OSA-related target organ damage. The impairment of mitochondrial oxidative phosphorylation during hypoxia, subsequently followed by reoxygenation, induces the production of reactive oxygen species (ROS) and the uncontrolled phosphorylation of numerous proteins (Figure 1). ROS generation involves the mitochondrial respiratory chain and numerous enzyme complexes, including NADPH oxidase and xanthine oxidase (XO) [4]. ROS are capable of limiting NO bioavailability by reacting with NO synthase (NOS) cofactors such as tetrahydrobiopterin. Superoxide can easily react with NO itself, creating a highly toxic peroxynitrite (ONOO^−^), a source of nitrosative stress leading to post-translational modifications of numerous proteins (nitration and S-nitrosylation) [5]. As far as the literature is concerned, it has been shown that the expression of superoxide dismutase (SOD), a key antioxidant enzyme, is significantly limited in subjects with OSA [6], whereas malondialdehyde (MDA), a commonly investigated marker of lipid peroxidation, is significantly increased in the course of OSA [7].

Alterations in the nitric oxide (NO) metabolic pathway and subsequent impairment of endothelial vasodilative function may play a pivotal role in the pathogenesis of increased cardiovascular risk in OSA. NO is not only an important vasodilator, but it also reduces platelet degranulation and decreases platelet and leukocyte adhesion to the endothelium [8]. Asymmetric dimethylarginine (ADMA) is the most important competitive inhibitor of nitric oxide synthase (NOS), thus regulating the bioavailability of NO. Elevated plasma levels of ADMA have already been shown to be an early predictor of increased cardiovascular risk [9]. ADMA is present not only in plasma but also in the intracellular compartment, including endothelial cells and human erythrocytes [10]. Erythrocytes, due to their high contribution to the hematocrit, could play an important role in the storage and accumulation of ADMA [11], thus modulating the plasmatic concentration and finally endothelial function by blocking nitric oxide synthase. ADMA is a highly regulated molecule, both in terms of its biosynthesis, degradation, metabolism, and transport. Its biosynthesis in intraerythrocytic cells is possible by protein-arginine methyltransferases (PRMTs) catalyzing the methylation of proteinic L-arginine [12]. RBCs contain arginine-rich proteins, derived mostly from lysis of the nucleus during erythropoiesis, which could be a potential source of inhibitory methylarginines [12]. Interestingly, increased methylation of the L-arginine residue by PRMT has been shown to be increased in hypoxia [13]. In vitro, lysis of erythrocytes results in the release of pathologically relevant quantities of free ADMA, which after 2 h of incubation increased sevenfold [14]. Nevertheless, another study suggests that in vivo hemolysis is unlikely to significantly increase human plasma concentrations of free ADMA [15].

Furthermore, the degradation of ADMA by the dimethylarginine dimethylaminohydrolases (DDAH1 and DDAH2) was decreased in the animal models of hypoxia [16]. Interestingly, the expression of the DDAH enzyme, was also identified in human RBCs [17,18].

Noteworthy, in addition to the cellular production and degradation of ADMA, the transmembrane transport of ADMA by CATs (cationic amino-acid transporters) determines its intracellular concentration. The transport of arginine and ADMA was examined in animal models and cell cultures. CAT-1, CAT-2A, CAT-2B, and the system y + L amino acid transporters were shown to be more relevant for transmembrane transport of arginine and ADMA [19]. CAT-1 expression is involved in erythroid hematopoiesis through importing L-arginine, which appears to be essential for the differentiation of RBCs [20]. CAT-1 is expressed in all tissues and organs except for the liver, where CAT-2A is expressed strongly and s the quantitative uptake of L-arginine and ADMA at high concentrations [19]. CAT-2B is induced by pro-inflammatory cytokines in numerous tissues, along with arginase and NOS [21]. In its physiological concentration range, ADMA is unlikely to impair CAT1-mediated transport of L-arginine. Conversely, high L-arginine concentrations inhibit the CAT1-mediated cellular uptake of ADMA [22].

Recently, it has been shown that increased cardiovascular morbidity in OSA patients could be related to the functional and structural changes in erythrocytes [23]. The red blood cells (RBCs) express a nitric oxide synthase (RBC-NOS, structurally identical with the endothelial isoform—eNOS), which, by producing NO, modifies the erythrocytes’ deformability through direct S-nitrosylation of cytoskeleton proteins, including spectrin-α and spectrin-β [24]. ADMA, by limiting NO production, may decrease the membrane fluidity of RBCs, suggesting that ADMA might have a close correlation with the rheologic behavior of erythrocytes in the microcirculation [25].

Hence, the goal of our study was to verify if ADMA concentrations within RBCs depend on the severity of OSA. Secondly, we aimed to verify if erythrocytes could play a role in the storage and accumulation of asymmetric dimethylarginine (ADMA) in OSA patients. The correlations between the erythrocyte nitric oxide metabolic pathway intermediates and the other risk factors and endothelial vasodilative function at the level of microcirculation were also investigated.

## 2. Materials and Methods

### 2.1. Bioethics Approval

The study protocol has been approved by the Bioethics Committee of Wroclaw Medical University: protocol nr KB-335/2018 from 29 May 2018 for studies involving humans. All the volunteers agreed to participate in this project by signing a written informed consent on the form previously verified by the Bioethics Committee. The project procedures are consistent with the principles of the Declaration of Helsinki (Seventh Revision, 64th World Medical Association meeting, Fortaleza, Brazil, 2013).

### 2.2. Subject Recruitment

Subjects suspected of OSA who were admitted to the hospital between January 2019–November 2020 for a polysomnography examination were considered for participation in this study. The initial inclusion criteria for these subjects were: suspicion of OSA based on signs and symptoms assessed by questionnaires (Berlin, Germany, STOP-BANG, Epworth scale); age between 30–70 years; and written informed consent. The exclusion criteria were the presence of diabetes, developed vascular disease such as past stroke, past myocardial infarction, previous revascularization, anemia, polycythemia, and malignancies. Subjects with a history of premature cardiovascular death in family history (men < 55 years old, women < 65 years old), comorbidity with rheumatic diseases, and taking drugs such as anticoagulants, antiplatelets, anti-inflammatory, immunosuppressants, as well as nebivolol (due to interference with the nitric oxide metabolism), were also excluded. After a careful analysis, a total of 47 subjects were enrolled in this study, but one subject was excluded because of untreated newly diagnosed diabetes mellitus.

### 2.3. Measurements

About 40 mL of peripheral venous blood was collected in the morning for the fasting condition, directly after the polysomnographic examination. In the hospital laboratory, we provided biochemical blood tests assessing cardiovascular risk, which were performed ad hoc from the obtained serum samples. The samples for biochemical analysis of selected intermediates involved in the nitric oxide biotransformation pathway were prepared simultaneously and stored at −80 °C until subsequent analyses.

Assessment of endothelial vasodilatory function using Laser Doppler Flowmetry (LDF) was performed in both groups, as described below.

One night, polysomnography was performed using the NOX-A1 system (Nox Medical, Reykjavik, Iceland). Polysomnograms were described following the standard sleep criteria introduced by the American Academy of Sleep Medicine Task Force [26].

Abnormal respiratory events were scored from the standardized airflow signal:

- apneas—defined as the absence of airflow for ≥10 s,
- hypopneas (a reduction in the amplitude of breathing by ≥30% for ≥10 s with a ≥3% decline in blood oxygen saturation SpO_2_) or followed by arousal.

### 2.4. Subgroups

After polysomnographic examination, the patients were divided into groups depending on the severity of their sleep apnea, based on the calculated apnea-hypopnea index (AHI) [27]. Patients with moderate and severe sleep apnea for whom CPAP treatment was indicated and recommended were collected in the OSA group. The patients with mild OSA as well as those with excluded OSA following the diagnostics formed the control group, without CPAP treatment. Such selection of the control group allowed for the matching of clinically similar patients, initially suspected with OSA, where the PSG result was the only one criterion assigning the subjects to a particular subgroup.

**Figure 2:**
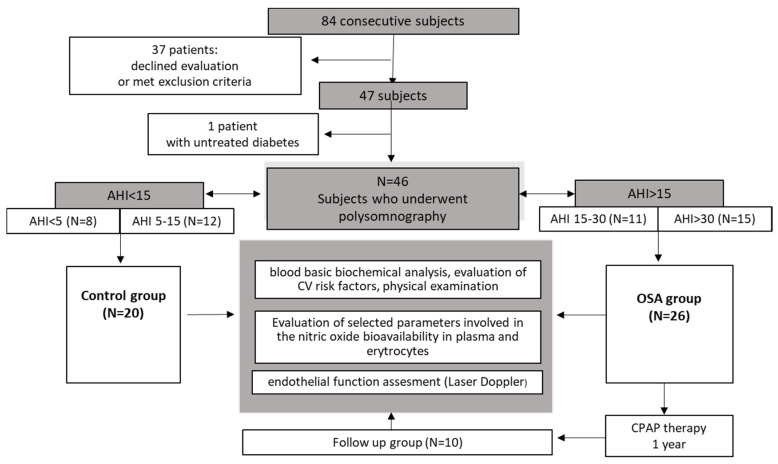
A flow chart of the study protocol. Abbreviations: OSA: Obstructive sleep apnea; AHI: apnea hypopnea index; CV: cardiovascular; CPAP: continuous positive airway pressure.

From the OSA group, only these subjects with compliance to the therapy and willingness to participate in the follow-up were invited for the second evaluation, one year after the diagnosis and beginning of the treatment (Figure 2).

### 2.5. Biochemical Analysis

The blood was collected using an S-Monovettes, (SARSTEDT, Sarstedt Germany). Whole blood for RBC isolation was drawn into the tube containing sodium citrate at a ratio of 1:10 (one part citrate to nine parts blood). The erythrocytes were obtained by removing the plasma and buffy coat of the blood by centrifugation at 1000× *g* for 10 min at room temperature and subsequently washing four-times with phosphate buffered saline (PBS). RBC count as well as contamination by leucocytes (WBC) and platelets (PLT) were assessed using the Sysmex XT-2000i. Purified cells were finally adjusted to a concentration of 2.5 × 10^9^ in the sample and stored at −80 °C until subsequent analyses. Each sample contained the same number of cells: 2.5 × 10^9^ erythrocytes/sample. As a result, all measurements of the intra-erythrocyte metabolites were conducted in the same number of pooled-down RBCs.

RBC metabolites extraction: RBC samples were thawed on ice, and afterwards, 10 µL of an internal standard solution and 1200 µL of a cold extraction solution of methanol, acetonitrile, and water (5:3:2) were added and vortexed (for 15 min, at 1200× *g*, at 4 °C). Samples were then centrifuged (15 min at 2200× *g*, at 4 °C) and the supernatants were transferred into the microcentrifuge tubes. Afterwards, the samples were dried at 50 °C.

The derivatization of amino acids was performed using benzoyl chloride (BCl) as reagent. Dried samples were dissolved in 100 µL of water and vortexed (5 min, 1200× *g*, 25 °C). Subsequently, 50µL of borate buffer (0.025 M Na_2_B_4_O_7_ × 10 H_2_O, 1.77 mM NaOH, pH = 9.2), 400 µL of acetonitrile, and 10 µL of 10% BCl in acetonitrile were added and vortexed again (10 min, 1200× *g*, 25 °C). Following this procedure, the samples were dried at 45 °C with a SpeedVac-Vacuum-Concentrator and subsequently reconstituted in 50 µL of a 3% solution of methanol in water and centrifuged (10 min at 15,000× *g*, 4 °C). The supernatant was transferred into a chromatographic polypropylene vial with 200 µL of insert.

The liquid chromatography–mass spectrometry analysis was conducted using the SYNAPT-G2 Si mass spectrometer, which was coupled with an Acquity I-Class ultra-performance liquid chromatography system (Waters, Milford, MA, USA). The mass spectrometer contained an electrospray ionization source. Data was acquired by a MassLynx 4.1 software (Waters) for the following ions (*m*/*z*): 237.1239, 243.1339, 263.1090, 267.1382, 279.1457, 286.1897, 307.1770, and 314.2209 for, ornithine, D6-ornithine, citrulline, D4-cytrulline, arginine, D7-arginine, ADMA, SDMA, and D7-ADMA, respectively.

The plasma concentrations of the NO-pathway metabolites were measured, as previously described by Fleszar et al. 2018 [28]. Afterwards, the samples were centrifuged (for 7 min, at 4 °C, using 22,000× *g*) and 100 µL of supernatant was diluted 4× with water, transferred to chromatographic glass vials for subsequent analysis, which was performed using the equipment and methods described above.

### 2.6. Endothelial Function Assessment

**Figure 3:**
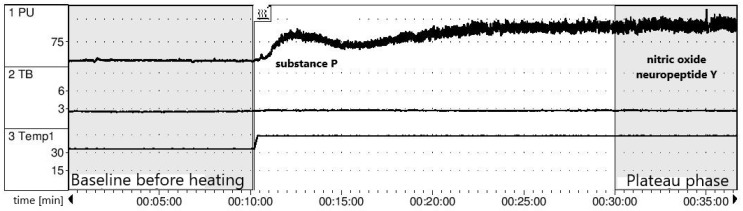
The Laser Doppler Flowmetry (LDF). Abbreviations: PU: perfusion unit; TB: total backscatter (the amount of light returned to the detector), Temp1: temperature of the probe 33–44 °C.

The NO-dependent vasodilatory function of the endothelium was measured by Laser Doppler Flowmetry (LDF), which enables the dynamic measurement of changes in the superficial microcirculation following application of a local thermal stimulus. Standard recording in the course of local heating consists of two main phases: the peak phase (lasting a few minutes), which depends on the stimulation of local sensory nerves leading to substance P excretion, and the plateau phase (after 20 min), conditioned mostly by nitric oxide but also by noradrenaline and neuropeptide Y [29]. The probe of the laser doppler flowmeter (Periflux 5000, Perimed, Järfälla, Sweden) was located on the forearm skin without any visible superficial vasculature. The studied arm was immobilized using a vacuum pillow, following the manufacturer’s recommendations. After 10 min of baseline recording at 33° C, heating was increased to 44° C for the next half hour. In order to prevent the effect of the baseline flow variability, the results are presented as the hyperemia index (HI = perfusion following 20min of heating divided by the perfusion before heating) (Figure 3).

### 2.7. Statistical Analysis

The differences between two continuous variables were assessed using the Mann–Whitney U-test or a Student’s *t*-test, as appropriate. The Wilcoxon test was used to compare paired groups before and after CPAP, as appropriate. Additionally, Spearman’s rank correlation coefficient was performed. The data is presented as the mean with the standard deviation (SD) or the median with the interquartile range (Q_1_–Q_3_), dependending on the normality of the distribution and variance homogeneity, previously assessed using the Shapiro Wilk test and Levene’s test, as appropriate.

All calculations were conducted with the Statsoft^®^ Statistica 13.3 software, Krakow, Poland, and GraphPad PRISM (9.2.0, San Diego, CA, USA).

## 3. Results

### 3.1. Baseline Characteristics

In the study healthy, controls (AHI < 5) were grouped together with the mild obstructive sleep apnea patients (AHI < 15) and compared to the group with moderate and severe OSA (AHI > 15). There were no significant differences regarding the age and sex distribution between the two groups. However, there were significant differences between the groups regarding weight, body mass index, white blood cells, CRP, uric acid, and insulin, together with HOMA-IR and QUICKI. (Table 1).

### 3.2. Assessment of Baseline Endothelial Function

No significant differences in endothelial function measured by the LDF were noted. Nevertheless, the hyperemia index reached the lowest average values in non-treated OSA subjects (11.2 ± 5.9 in the control vs. 9.6 ± 5.3 in OSA before CPAP), respectively, *p* > 0.05 for each analysis (Table 2).

### 3.3. Parameters of Nitric Oxide Bioavailability in Erythrocytes and Plasma

**Figure 4:**
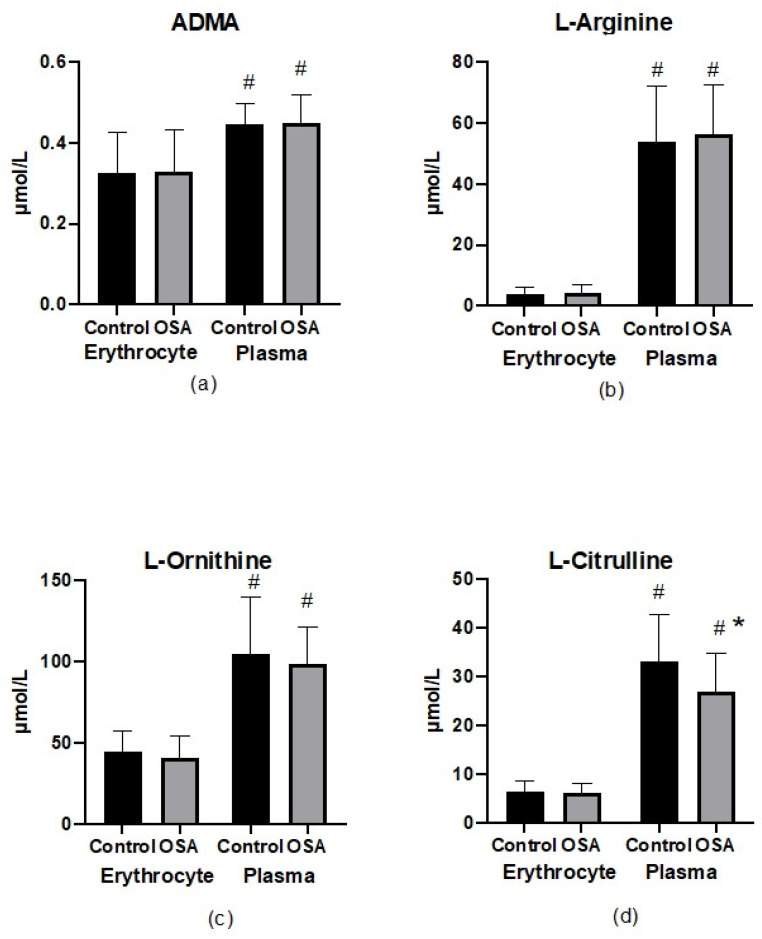
(**a**–**d**) Nitric oxide metabolite concentrations in different compartments. Bars and whiskers represent the mean ± SD. Abbreviations: ADMA: asymmetric dimethylarginine; OSA: obstructive sleep apnea. * denotes *p* < 0.05 vs. control in the same compartment; # denotes *p* < 0.05 between compartments in the same group.

As shown in Figure 4, no significant differences regarding metabolites of the NO pathway were found apart from the plasma citrulline concentration, which was lower in patients with OSA (26.9 ± 7.4 vs. 33.1 ± 9.4 μM, *p* < 0.05). All of the altered nitric oxide metabolites were identified at higher concentrations in plasma than in the erythrocyte compartment, which was most significantly pronounced for the arginine concentrations. The erythrocyte-ADMA concentration was lower than in plasma irrespective of the presence of OSA (0.33 ± 0.12 vs. 0.45 ± 0.08 μM in OSA, *p* < 0.05 and 0.33 ± 0.1 vs. 0.45 ± 0.07 μM in the control, *p* < 0.05).

**Figure 5:**
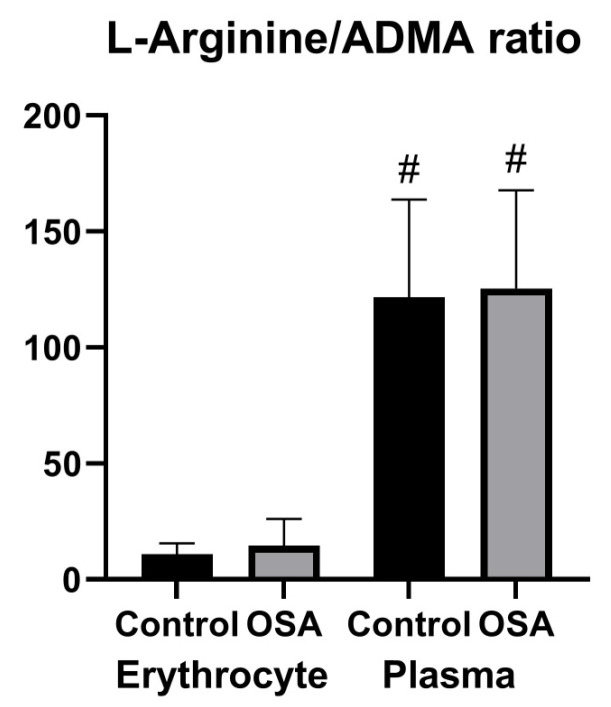
The NO synthase substrate (arginine) to competitive inhibitor (ADMA) ratio in plasma and erythrocytes. Bars and whiskers represent the mean ± SD. Abbreviations: ADMA: asymmetric dimethylarginine; OSA: obstructive sleep apnea. # denotes *p* < 0.05 vs. the erythrocyte compartment in the same group.

The substrate/inhibitor ratio of nitric oxide synthase in both the plasma and erythrocyte compartments was also assessed (Figure 5). The difference between the plasma and erythrocyte nitric oxide bioavailability measured by the arginine/ADMA ratio was mainly determined by changes in the arginine concentration. There were no differences in the arginine/ADMA ratio in both groups; nevertheless, the arginine/ADMA ratio was about 10-fold lower in the erythrocyte compartment than in the plasma.

### 3.4. Effect of CPAP

**Figure 6:**
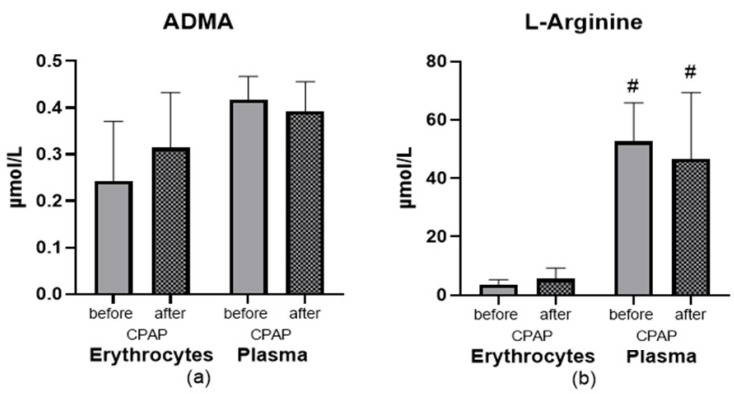
(**a**,**b**). The effect of 1-year CPAP therapy on the bioavailability of NO metabolites. Bars and whiskers represent the mean ± SD. Abbreviations: ADMA: asymmetric dimethylarginine; OSA: obstructive sleep apnea. # denotes *p* < 0.05 between compartments in the same group.

Subjects from the OSA group who were provided with CPAP therapy and were using it regularly for about one year were asked for a second evaluation. Ten subjects met the criteria and agreed to the examination. CPAP therapy after one year of use did not show any significant changes in the bioavailability of NO metabolites’ (Figure 6).

**Figure 7:**
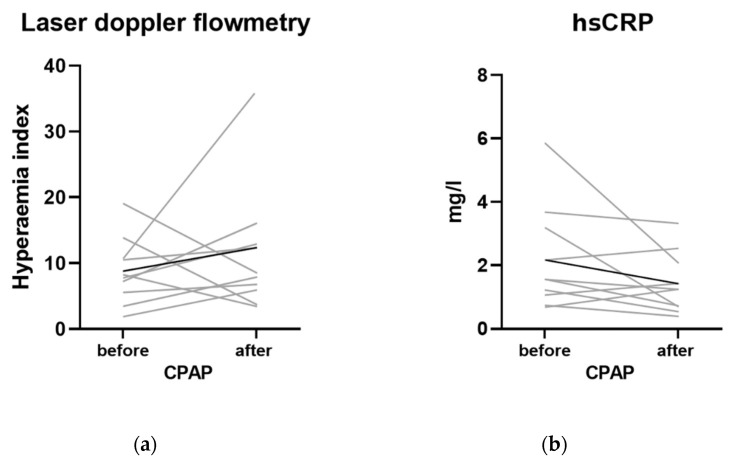
(**a**,**b**) The effect of 1-year CPAP therapy on hsCRP and endothelial function measured by laser doppler flowmetry.

There was a trend for CPAP towards lowering hsCRP (Figure 7 and Table S1). Nonetheless, the hyperemia index reached greater average values in OSA subjects following CPAP. (9.6 ± 5.3 in OSA before CPAP and 11.64 ± 3.97 in OSA after 1-year CPAP, respectively, *p* > 0.05).

## 4. Discussion

Recent findings postulate a pivotal role for endothelial dysfunction in OSA as the first step leading to the development of target organ damage [30]. Nevertheless, the data from human studies on this matter is scarce. Moreover, the role of erythrocytes in maintaining the bioavailability of NO has yet to be proven. This is the first prospective study that evaluated the NO metabolic pathway in both plasma and erythrocyte compartments in patients with obstructive sleep apnea (OSA) as well as following 1-year CPAP treatment. Furthermore, endothelial function, assessed by laser doppler flowmetry and reflecting the vasodilative NO-dependent function of microcirculation, was measured in OSA subjects at baseline and prospectively following CPAP treatment.

### 4.1. NO Biotranformation in Erythrocytes and Plasma in OSA and Non-OSA Subjects

Some studies have identified a cross-talk between the occurrence of OSA and high plasma ADMA levels [31], which is not influenced by the presence or absence of conventional risk factors [32]. In our study, the plasma ADMA concentrations were similar in both groups, which has been previously demonstrated [33].

The levels of ADMA in our study measured in plasma of about 0.5 μmol/L may be below those required for competitive NOS inhibition, as the concentrations of its substrate, L-arginine, are manyfold above the Km for NOS [34]. In our study, the average concentrations of intraerythrocytic ADMA were even lower—about 0.3 μmol/L. RBC could play a more important role in the storage pool of ADMA. Interestingly, in patients undergoing hemodialysis, the urea and creatinine had a rebound ratio measured by an increase after 1 h of hemodialysis of less than 10%. In contrast, a considerable rebound ratio of 30% of ADMA was simultaneously detected [35]. Hence, we assume that ADMA may present a multicompartmental distribution in which RBCs could play a pivotal role, including in subjects with OSA.

**Figure 8:**
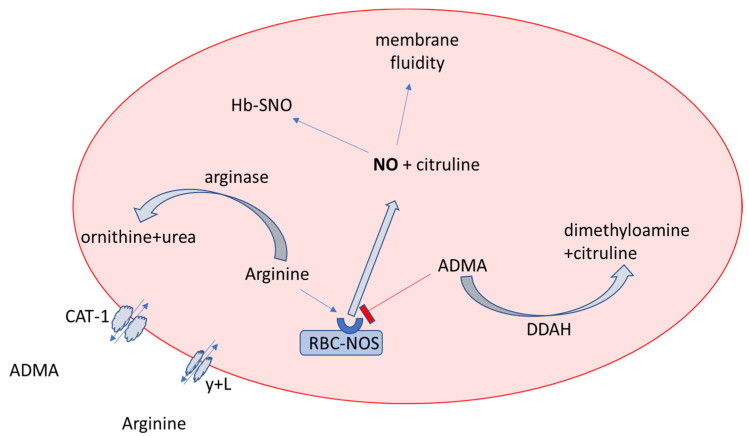
Nitric oxide bioavailability in erythrocytes. Abbreviations: NO: nitric oxide; RBC-NOS: nitric oxide synthase; ADMA: asymmetric dimethylarginine; CAT-1: cationic amino acid transporter-1; HB-SNO: S-nitrosohemoglobin; DDAH: dimethylarginine dimethylaminohydrolase; y + L: amino acid transporter.

RBCs contain eNOS and display NO-like bioactivity [36]. Noteworthy, in our study the concentration of L-arginine in RBCs was about 10-fold lower than in plasma, resulting in a lower L-arginine/ADMA ratio and lower substrate bioavailability for NOS, which is confirmed by the lower citrulline level (a side product of the NO synthesis). The L-arginine concentration is under tight control by arginase 1 (Figure 8), whose activity is regulated during ischemia-reperfusion/hypoxia-reoxygenation injuries [36]. The up-regulation of arginase activity could be caused by the peroxynitrite donor in RBCs [37]. Diminished efflux of arginine from plasma to erythrocytes is another potential cause of 10-fold lower arginine concentrations in RBCs than in plasma.

The role of RBC-NOS in supporting NO production and maintaining endothelial function remains uninvestigated. Only a small amount of the nitric oxide carried by the Hb is released from the RBCs, but the transition from high to low oxygen tension in the peripheral vasculature enhances its release as SNO-based vasodilatory action [38].

### 4.2. Effect of CPAP

Interestingly, in a follow-up study 1-year after CPAP treatment, there was a tendency for higher concentrations of arginine in erythrocytes. This could be partially explained by the lower activity of arginase 1, because of less ROS production, which increases arginase 1 activity. Together with intraerythrocytic arginine, increased its competitive inhibitor ADMA, the Arginine/ADMA ratio was maintained at a similar level. A one-year treatment with CPAP also showed decreased CRP levels. Another study suggests that a lowered CRP might be a valuable predictor of success in OSA treatment monitoring [39].

### 4.3. L-Citrulline and NO Production

Citrulline is efficiently recycled in endothelial, immune, and kidney cells and is easily converted into arginine [40]. On the other hand, citrulline is a side product in the reaction catalyzed by the eNOS as well as in the ADMA degradation, catalyzed by the DDAH, which might also prevent excessive and uncontrolled NO synthesis [40]. In our study, plasma citrulline concentrations were lower in OSA patients than in controls, which could be the first sign of diminished nitric oxide synthesis.

### 4.4. Other Differences in the Demographic and Biochemical Characteristics between the Groups

The comparison of groups with AHI < 15 and mild OSA with AHI > 15 showed differences in some anthropometric and metabolic parameters, including BMI, weight, fasting insulin, leukocytes, uric acid, and CRP values. Noteworthy, metabolic syndrome and all its components constitute the risk factors for OSA development, and as a result they do coexist commonly with OSA [41]. Obesity and insulin resistance, synergistically with OSA, could multiply the cardiovascular risk by increasing oxidative stress, promoting inflammation and apoptosis [42] and endothelial dysfunction, which at the early stages of atherogenesis in clinically healthy subjects might be reversible [43]. In fact, impairment of the availability of NO could multiply the endothelial dysfunction in the comorbidity of OSA and pre-diabetes. Therefore, a subgroup analysis of the control group including only “metabolically-matched” subjects to the OSA group could provide detailed information on the direct effect of OSA on endothelial function and the NO metabolites. Nevertheless, similar values of the reactive hyperemia index (RHI), reflecting the endothelial vasodilative function of microcirculation as well as the plasma and erythrocyte levels of the NO metabolites between the groups, do not indicate that the presence of biochemical and demographical differences could blur the effect of OSA and its treatment in the study group.

## 5. Study Limitations

The subjects enrolled in this study suspected of having OSA had concomitant cardiovascular risk factors, such as glucose intolerance, hypertension, obesity, and smoking; therefore, the impact of OSA severity could be interfered with. Furthermore, the erythrocytes contain transmembrane proteins, whose function may affect compartment distribution and study outcomes. Nevertheless, rapid, ad hoc isolation of erythrocytes collected from the blood drawn and subsequent freezing of the RBC samples at −80 °C limited these phenomena. The small population of subjects enrolled in the study is another limitation.

## 6. Conclusions

The erythrocyte pool of the intermediates of the NO metabolic pathway does not depend on the severity of OSA, and erythrocytes could constitute a high-volume buffer in their storage in OSA patients. The use of CPAP for one year did not result in changes in the balance between the erythrocyte and the plasmatic pool of the NO metabolic pathway intermediates. The results from this prospective study are a step forward in understanding the role of the erythrocyte compartment and the intra-erythrocyte pathways regulating the NO bioavailability and paracrine endothelial function in the hypoxia-reoxygenation setting, such as obstructive sleep apnea. Nevertheless, future large prospective studies with precise matching of the cases in groups regarding comorbidities would limit the potentially distracting effect of concomitant disorders on the results.

## Figures and Tables

**Table 1:** The baseline characteristics of groups including cardiovascular risk stratification. The results are presented as mean ± SD or median ± (Q1–Q3).

Parameter	Control Group AHI < 15 *n* = 20	OSA Group AHI > 15 *n* = 26	*p*-Value
	(Mean ± SD) or Median(Q1–Q3)	(Mean ± SD) or Median (Q1–Q3)	
Men (n, %)	13 65%	24 92%	NS
Women (n, %)	7 35%	2 8%	NS
Age (years)	50.6 ± 7.41	50.6 ± 9.95	NS
Height (cm)	173.6 ± 11.7	174.9 ± 6.93	NS
Weight (kg)	79.2 ± 17.4	92.0 ± 16.2	<0.05
Hypertension (n, %)	4.0 20%	11.0 42%	NS
BMI (kg/m^2^) median(Q1–Q3)	25.4 (23.7–28.2)	29.1 (26.4–33.1)	<0.05
RBC (mln/µL)	5.04 ± 0.36	5.05 ± 0.34	NS
WBC (k/µL) median(Q1–Q3)	5.66 (4.41–6.91)	6.66 (5.22–8.98)	<0.05
Hb (g/dL)	15.2 ± 1.13	15.3 ± 1.00	NS
PLT (k/µL)	233.7 ± 45.0	243.0 ± 62.6	NS
Ht (%)	44.8 ± 3.14	45.5 ± 2.61	NS
MCV (fL)	88.9 ± 2.53	90.2 ± 4.01	NS
MCH (pg)	30.2 ± 1.11	30.3 ± 1.33	NS
MCHC (g/dL)	34.0 ± 0.83	33.6 ± 0.91	NS
HbA1c (%)	5.52 ± 0.36	5.58 ± 0.38	NS
ALT (U/L) median(Q1–Q3)	25.0 (19.5–41.5)	33.5 (22–39)	NS
LDL (mg/dL)	129.3 ± 35.7	140.0 ± 27.6	NS
Total cholesterol (mg/dL)	212.1 ± 36.9	224.6 ± 39.9	NS
HDL (mg/dL)	57.9 ± 11.3	51.6 ± 12.7	NS
Triglycerides (mg/dL) median(Q1–Q3)	124.6 (86–145)	164.9 (116–208)	NS
TSH (µlU/mL) median(Q1–Q3)	1.46 (1.04–1.64)	1.32 (0.9–1.8)	NS
Creatinine (mg/dL) median(Q1–Q3)	0.97 (0.86–1.04)	0.99 (0.89–1.08)	NS
eGFR (mL/min/1.73 m^2^)	80.5 ± 11.8	86.5 ± 14.8	NS
Uric acid (mg/dL)	4.95 ± 1.05	6.38 ± 0.92	<0.05
Urea (mg/dL)	30.3 ± 6.76	31.5 ± 7.53	NS
Mg (mmol/L)	2.13 ± 0.11	2.12 ± 0.14	NS
K (mmol/L)	4.35 ± 0.29	4.28 ± 0.28	NS
Na (mmol/L)	140.4 ± 1.83	141.5 ± 1.86	NS
hsCRP (mg/L) median(Q1–Q3)	0.46 (0.28–0.73)	1.43 (0.80–3.45)	<0.05
Ca (mmol/L)	9.37 ± 0.35	9.34 ± 0.27	NS
Glucose (mg/dL)	94.9 ± 10.4	100.5 ± 9.80	NS
Insulin (µU/mL) median(Q1–Q3)	6.15 (5.0–7.3)	11.9 (6.4–14.4)	<0.05
HOMA-IR median(Q1–Q3)	1.48 (1.16–1.64)	2.93 (1.36–3.78)	<0.05
QUICKI median	0.36 ± 0.03	0.33 ± 0.03	<0.05
AHI (events/h) median(Q1–Q3)	6.1 (3.0–8.3)	36.4 (20–37)	<0.05
ODI (events/h) median(Q1–Q3)	5.0 (2.2–9.0)	34.4 (20–39)	<0.05
Mean saturation (%)	94.3 ± 1.2	92.5 ± 1.7	<0.05
Duration of desaturation < 90% (% of total sleep time) median(Q1–Q3)	0.1 (0.0–0.9)	5.1 (1.6–11.0)	<0.05

Abbreviations: NS: result not statistically significant; BMI: body mass index; RBC: red blood cells; WBC: white blood cells; MCV: mean (red blood) cell volume; MCH: mean corpuscular hemoglobin; MCHC: mean corpuscular hemoglobin concentration; PLT: platelets; eGFR: estimated glomerular filtration rate; HbA1c: glycated hemoglobin; HOMA-IR: homeostatic model assessment of insulin resistance; QUICKI: quantitative insulin sensitivity check index. HDL: high-density lipoprotein; LDL: low-density lipoprotein; hsCRP: high-sensitivity C-reactive protein; TSH: thyroid-stimulating hormone; AHI: apnea-hypopnea index; ODI: oxygen desaturation index; Hb: hemoglobin; Ht: hematocrit.

**Table 2:** Assessment of endothelial function by laser-doppler-flowmetry (LDF).

	Hyperemia Index
	Mean ± SD
Control group	11.2 ± 5.9
OSA group before CPAP	9.6 ± 5.3
OSA after 1 year of CPAP	11.6 ± 4.0

## Data Availability

The original data used to support the findings of this study are available upon request from the corresponding author.

## Supplementary Materials

The following supporting information can be downloaded at: https://www.mdpi.com/article/10.3390/ijerph192214719/s1, Table S1: Baseline characteristics of subjects before and after 1 year of CPAP.
