# Supplementary material for: Effect of Obstructive Sleep Apnea and CPAP Treatment on the Bioavailability of Erythrocyte and Plasma Nitric Oxide
Source: Int J Environ Res Public Health. 2022 Nov 9;19(22):14719. doi: 10.3390/ijerph192214719 (PMC9690918; doi:10.3390/ijerph192214719)
Supplement: Supplementary file 1 [file ijerph-19-14719-s001.zip › ijerph-1959685-supplementary.pdf]

Supplementary Table S1. Baseline characteristics of subjects before and after 1 year of CPAP.

Parameter	OSA group before CPAP treatment Mean ± SD			OSA group after 1 year of CPAP treatment Mean ± SD			*p* value
Age [years]	54.90	±	7.52	55.90	±	7.50	p<0.05
BMI [kg/m^2]	28.22	±	2.72	28.48727	±	2.587217	NS
WBC [k/μL]	7.21	±	1.91	7.00	±	3.34	NS
RBC [mln/μL]	5.19	±	0.34	5.08	±	0.27	NS
Hb [g/dL]	15.56	±	0.81	15.27	±	0.73	NS
Ht [%]	46.39	±	2.29	44.66	±	2.08	NS
MCV [fL]	89.52	±	3.83	87.97	±	4.20	NS
MCH [pg]	30.00	±	0.75	29.96	±	1.27	NS
PLT [k/μL]	212.30	±	41.31	230.10	±	61.81	NS
HbA1c [%]	5.63	±	0.35	5.74	±	0.63	NS
AlT [U/L]	28.10	±	7.00	25.75	±	2.22	NS
LDL [mg/dL]	133.10	±	24.80	113.80	±	32.55	NS
Total cholesterol [mg/dL]	217.80	±	35.85	192.67	±	35.52	NS
HDL [mg/dL]	52.20	±	17.09	47.67	±	12.11	NS
Triglycerydes [mg/dL]	162.40	±	80.60	166.67	±	145.42	NS
hsCRP [mg/L]	4.58	±	7.73	2.65	±	3.54	NS
TSH [μLU/mL]	1.72	±	1.13	1.06	±	0.28	NS
Creatinine [mg/dL]	1.07	±	0.19	1.01	±	0.13	NS
eGFR [mL/min/1,73m^2]	79.00	±	15.53	83.17	±	13.08	NS
Uric acid [mg/dL]	6.69	±	1.16	6.37	±	0.98	NS
Urea [mg/dL]	35.70	±	9.44	33.17	±	4.07	NS
Mg [mmol/L]	2.15	±	0.11	1.98	±	0.12	NS
K [mmol/L]	4.24	±	0.26	4.26	±	0.24	NS
Na [mmol/L]	141.90	±	1.66	141.00	±	1.41	NS
Ca {mmol/L]	9.28	±	0.17	9.55	±	0.15	NS
Glucose [mg/dL]	101.40	±	9.99	103.33	±	11.68	NS
InsμLin [uU/mL]	9.47	±	5.37	10.71	±	4.95	NS
HOMA-IR	2.40274	±	1.37291	2.85701	±	1.631927	NS
AHI [events/h]	34.12	±	14.11	2.55000	±	2.05061	p<0,05
ODI [events/h]	33.10	±	13.81	2.61000	±	2.10050	p<0,05
Mean Saturation [%]	92.86	±	1.50	94.00000	±	2.01384	p<0,05
Desaturation <90% [% of total sleep time]	7.71	±	6.00	0.24000	±	0.20258	p<0,05

Abbreviations: NS: result statistically non-significant; BMI: body mass index; RBC: red blood cells; WBC: white blood cells MCV: mean [red blood] cell volume; MCH: mean corpuscular hemoglobin; MCHC: mean corpuscular hemoglobin concentration; PLT: platelets; eGFR: estimated glomerular filtration rate; HbA1c: Glycated hemoglobin; HOMA-IR: Homeostatic Model Assessment of Insulin Resistance; HDL: high-density lipoprotein; LDL: low-density lipoprotein; hsCRP: high-sensitivity C-reactive protein; TSH: thyroid-stimulating hormone; AHI: apnea-hypopnea index; ODI: oxygen desaturation index; Hb: hemoglobin; Ht: hematocrit.
